# Malnutrition in Japanese patients with chronic obstructive pulmonary disease: A prospective cohort study

**DOI:** 10.1097/MD.0000000000045203

**Published:** 2025-10-10

**Authors:** Seiichi Kobayashi, Manabu Ono, Masatsugu Ishida, Hikari Satoh, Masakazu Hanagama, Masaru Yanai

**Affiliations:** a Department of Respiratory Medicine, Japanese Red Cross Ishinomaki Hospital, Ishinomaki, Miyagi, Japan.

**Keywords:** body mass index, chronic obstructive pulmonary disease, exacerbation, Global Leadership Initiative on Malnutrition, malnutrition, mortality

## Abstract

Malnutrition is a common condition in patients with chronic obstructive pulmonary disease (COPD) and is associated with impaired lung function, worsened dyspnea, increased exacerbations, and higher mortality. However, diagnostic criteria for malnutrition have not been consistent. The Global Leadership Initiative on Malnutrition criteria were recently introduced to standardize the diagnosis of malnutrition. This study aimed to evaluate the prevalence, characteristics, and outcomes of malnutrition according to low body mass index of the Global Leadership Initiative on Malnutrition criteria in Japanese patients with COPD. A prospective observational study was conducted among Japanese patients with COPD from April 2018 to September 2024. Patient characteristics, exacerbation frequency, and mortality were assessed over a 5-year follow-up period. The odds ratios (ORs) for exacerbations were estimated using logistic regression analysis. A Cox proportional hazards model was used to examine the association between malnutrition and mortality. Among 309 patients (294 males, 15 females; median age, 76 years) with stable COPD, 14.5% were diagnosed with malnutrition. Patients diagnosed with malnutrition had a higher median age and showed greater airflow obstruction, more severe dyspnea, worsened health status, depressive state, muscle weakness, reduced exercise capacity, and lower physical activity compared with those who without malnutrition (all *P* < .05). Malnourished patients had a higher risk of exacerbations, including hospital admissions, compared with well-nourished patients (OR, 2.7, 95% confidence interval [CI], 1.2–5.9; and OR, 3.6, 95% CI, 1.5–8.7, respectively). All-cause and respiratory-associated mortality were significantly higher in malnourished patients than those without malnutrition (43.6% vs 19.1%, and 10.3% vs 2.5%, respectively; both *P* < .01). The adjusted hazard ratio for all-cause mortality in malnourished patients was 2.3 (95% CI, 1.3–4.2). Malnutrition is associated with adverse outcomes in Japanese patients with COPD, emphasizing the need for timely diagnosis and management.

## 1. Introduction

Chronic obstructive pulmonary disease (COPD) is the leading cause of morbidity and mortality worldwide, with an economic and social burden that is both substantial and increasing.^[1]^ COPD is a heterogeneous lung condition characterized by chronic respiratory symptoms and persistent, often progressive, airflow obstruction.^[1]^ Patients suffering from COPD manifest respiratory symptoms and often exhibit comorbid malnutrition.^[2,3]^ Recent meta-analyses estimate that ~30% of patients with COPD suffer from malnutrition.^[4]^ The causes of malnutrition in COPD include increased energy expenditure and reduced energy intake, which are influenced by factors such as tissue hypoxia; elevated resting metabolic rate; proinflammatory cytokines; feeding hormones; medications; anorexia; smoking; aging; and psychological, social, and environmental factors.^[2,3,5]^ Malnutrition and underweight are associated with impaired lung function, increased dyspnea, reduced exercise capacity, diminished quality of life, more frequent exacerbations, and higher mortality in COPD.^[3,6–8]^

Although malnutrition is a global concern associated with increased mortality, morbidity, and healthcare burden, the diagnosis of malnutrition has been challenging due to a lack of consensus on the diagnostic criteria for application in clinical setting. Recently, the Global Leadership Initiative on Malnutrition (GLIM) engaged the global nutrition communities to create a consensus on the criteria for malnutrition diagnosis, the GLIM criteria.^[9]^ The GLIM criteria utilize a 2-step approach: patients are first identified using a validated screening tool, followed by diagnostic assessment and grading of malnutrition severity (moderate or severe). The GLIM criteria have high diagnostic accuracy for distinguishing patients with malnutrition and have been used as a gold standard for diagnosing malnutrition in clinical practice.^[10]^ Assessment of nutritional status using the GLIM criteria may facilitate early detection and management of patients with stable COPD; however, only a limited number of studies have applied GLIM or other diagnostic criteria to COPD populations. Moreover, evidence from large-scale prospective studies remains lacking.

We aimed to clarify the prevalence, clinical characteristics, and outcomes of malnutrition according to low body mass index (BMI) in the GLIM criteria by analyzing prospectively collected data from a cohort of patients with stable COPD. In this study, malnutrition refers specifically to undernutrition as defined by the GLIM criteria.

## 2. Methods

### 2.1. Study design and setting

This study was part of an ongoing cohort study (UMIN Clinical Trials Registry identifier: UMIN000032112) aimed at evaluating health outcomes of patients with COPD in a community setting. We conducted an observational study analyzing prospectively collected data during consecutive scheduled visits of patients registered in the Ishinomaki COPD Network (ICON)^[11,12]^ between April 2018 and September 2024. All eligible cases recorded in the local registry during the study period were included in the analysis, and no formal sample size calculation was performed in advance.

The study adhered to the ethical principles of the Declaration of Helsinki and received approval from the Ethics Committee of Japanese Red Cross Ishinomaki Hospital (approval number: 12-14-1; approval date: April 21, 2015). All patients provided written informed consent prior to participation.

### 2.2. Participants

All participants were enrolled from the ICON registry^[11,12]^ between April 2018 and May 2019. ICON is a regional, multicenter, interdisciplinary collaboration system designed to provide comprehensive care for patients with COPD. It comprises 80 primary care practices and a 460-bed tertiary community hospital (Japanese Red Cross Ishinomaki Hospital) located in Ishinomaki and nearby cities in Japan. Patients registered in ICON received standard care in general practice clinics based on established guidelines.^[1,13]^ The patients also undergo scheduled examinations, educational sessions, and self-management intervention and then repeatedly receive an individually tailored program according to a comprehensive assessment at the Japanese Red Cross Ishinomaki Hospital every 6 to 12 months. Patients participating in an educational program were trained in early recognition of exacerbations and provided with a written action plan for managing exacerbations using a self-management diary. During exacerbations, patients are initially treated by their general practitioners and referred to the Japanese Red Cross Ishinomaki Hospital if necessary. Patients were prescribed short-acting bronchodilators but were not prescribed oral corticosteroids or antibiotics for self-administration during exacerbations.

Patients with stable COPD who were aged ≥40 years and had a smoking history of at least 10 pack-years were included in this study. The diagnosis of COPD was according to the global initiative for chronic obstructive lung disease (GOLD) criteria.^[1]^ Persistent airflow obstruction, defined as a post-bronchodilator forced expiratory volume in 1 second/forced vital capacity ratio of <0.7, was confirmed by repeated spirometry.

Exclusion criteria included: chronic bronchitis or emphysema without airflow obstruction, active tuberculosis, history of lung resection, neuromuscular disease, and active malignant disease. Patients who experienced COPD exacerbations in the 4 weeks preceding data collection were also excluded.

Participants underwent clinical and physiological evaluation at the Japanese Red Cross Ishinomaki Hospital at baseline and were followed up for 5 years or until death, whichever occurred first.

### 2.3. Clinical and physiological evaluation

Sociodemographic characteristics, smoking history, a history of exacerbations, and regular medication use were recorded for each patient. Comorbidities were assessed through patient interviews and medical records and were classified into categories including cardiovascular disease, cerebrovascular disease, diabetes, gastroduodenal ulcer or gastroesophageal reflux, and osteoporosis.

Body weight and height were measured using standardized protocols. BMI was calculated as body weight in kilograms divided by the square of height in meters. The annual body weight change was calculated by subtracting the initial weight at the baseline from the final weight and dividing the result by the number of follow-up years. The percentage change in body weight was calculated as the weight change divided by the initial weight and multiplied by 100. Handgrip strength was assessed as described previously.^[12]^ Muscle weakness is defined as a handgrip strength of <28 kg in men and <18 kg in women according to the criteria of physical frailty.^[14]^

Dyspnea was evaluated using the modified Medical Research Council dyspnea scale.^[1,13]^ COPD-related health status was assessed using the COPD Assessment Test.^[1,13]^ Mental status was evaluated using the Hospital Anxiety and Depression Scale, with anxiety and depression subscale scores ranging from 0 to 21 and scores of ≥8 indicating clinically relevant symptoms, as described previouly.^[12]^

Pulmonary function tests were conducted by well-trained technicians while patients were stable, following established guidelines.^[15,16]^ The severity of airflow obstruction was classified according to GOLD criteria.^[1]^ The 6-minute walk test was performed following the ATS guidelines,^[17]^ with modifications including oxygen saturation monitoring and interruption of walking if oxygen saturation dropped below 85%.^[12]^

Physical activity was measured using a 3-axis accelerometer, and daily step counts were recorded as previously described.^[12]^

Blood samples were collected at baseline, and serum C-reactive protein levels were measured according to standard operating procedures at the Clinical Laboratory of the Japanese Red Cross Ishinomaki Hospital.

GOLD ABE grouping was performed according to the GOLD report.^[1]^

To minimize potential sources of bias, we applied standardized protocols for data collection and trained all investigators in the use of validated questionnaires and the study instruments.

### 2.4. Diagnosis of malnutrition

The GLIM criteria were applied to diagnose malnutrition.^[9]^ These criteria include at least 1 of 3 phenotypic components: low BMI, non-volitional weight loss, and reduced muscle mass; and at least 1 of 2 etiologic components: reduced food intake or assimilation and inflammation or disease burden. All participants in this study met the etiologic criterion, as COPD corresponds to the etiologic components of inflammation/disease burden. Malnutrition was diagnosed according to low BMI in the GLIM criteria, defined as <18.5 kg/m² for patients aged <70 years and <20 kg/m² for those aged ≥70 years.

### 2.5. Outcomes

An exacerbation of COPD was defined as an event characterized by increased dyspnea and/or cough and sputum.^[1]^ The exacerbation rate was evaluated through direct patient interviews, self-management diaries completed by patients or caregivers, referral letters from general practitioners, and medical record reviews. Exacerbations requiring antibiotics and/or systemic corticosteroids to manage respiratory symptoms were included in this study, while patients with mild exacerbations managed solely with short-acting bronchodilators were excluded.

Life status was monitored throughout the study period, and the cause of death was determined by reviewing medical records or conducting telephone interviews.

### 2.6. Statistical analysis

Data were presented as number (percentage), mean ± standard deviation, or median (interquartile range). Differences between the 2 groups were analyzed using Student *t* test or the Mann–Whitney *U* test. Fisher exact test was applied to determine whether the proportions of categories between the 2 groups differed significantly. The odds ratios for exacerbations were estimated using logistic regression analysis. Kaplan–Meier curves of cumulative survival were generated, and mortality rates between groups were compared using the log-rank test. A Cox proportional hazards model, adjusted for potential cofounding factors associated with mortality, was used to examine the association between malnutrition and mortality, presenting hazard ratios and 95% confidence intervals (CIs). All statistical analyses were conducted using EZR (Saitama Medical Center, Jichi Medical University, Saitama, Japan), a graphical user interface for R (The R Foundation for Statistical Computing, Vienna, Austria).^[18]^ Missing data were handled using a complete-case analysis. No imputation was performed, and only participants with available data for all variables of interest were included in the multivariable analyses. Participants who were lost to follow-up were excluded from the analysis of exacerbations and mortality. Statistical significance was set at *P* < .05.

## 3. Results

### 3.1. Patient characteristics

**Figure 1.**
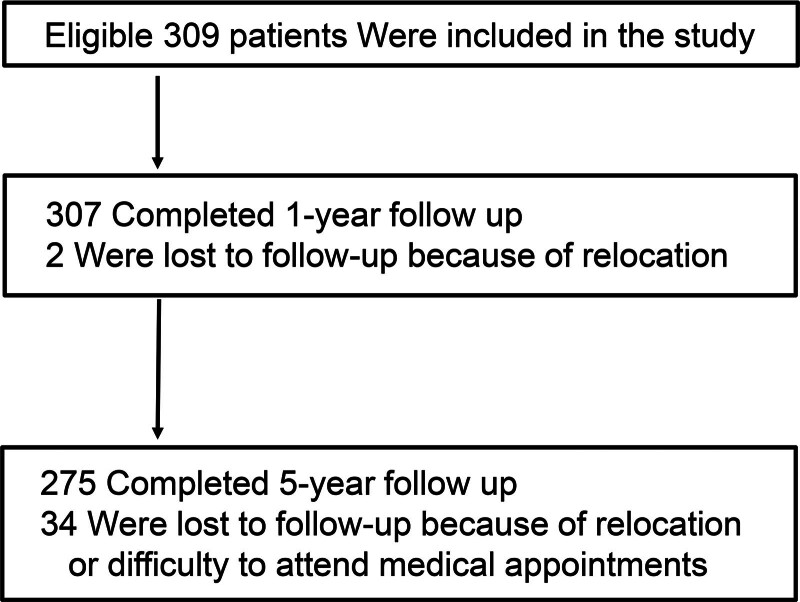
Patient inclusion flowchart.

A total of 309 eligible patients with stable COPD (294 males and 15 females; median age: 76 years) were included in the study (Fig. 1). Patient demographics and clinical characteristics are shown in Table 1.

**Table 1:** Patient demographics and clinical characteristics.

Characteristic	Total (N = 309)
Age, years	75 (70–80)
Age category, years	
40–64	21 (6.8)
65–74	116 (37.5)
75–84	147 (47.6)
≥85	25 (8.1)
Sex	
Male	294 (95.1)
Female	15 (4.9)
BMI, kg/m²	23.4 (21.6–26.0)
Smoking history, pack-years	52 (40–69)
Current smoking	13 (4.2)
FEV_1_, L	1.54 (1.08–1.91)
%FEV_1_, %	61.7 (45.7–77.8)
FVC, L	3.06 (2.60–3.56)
GOLD stage	
1	66 (21.3)
2	151 (48.9)
3	62 (20.1)
4	30 (9.7)
GOLD group	
A	207 (67.0)
B	72 (23.3)
E	30 (9.7)
Regular medication*	
LAMA	255 (82.5)
LABA	202 (65.4)
ICS	85 (27.5)
Long-term oxygen therapy	34 (11.0)
Comorbidity	
Cardiovascular disease	97 (31.4)
Cerebrovascular disease	19 (6.1)
Diabetes	46 (14.9)
Gastroduodenal ulcer or GERD	38 (12.3)
Osteoporosis	36 (11.7)

Data are presented as numbers (percentages) or medians (interquartile range).

BMI = body mass index, FEV_1_ = forced expiratory volume in 1 second, FVC = forced vital capacity, GERD = gastroesophageal reflux disease, GOLD = global initiative for chronic obstructive lung disease, ICS = inhaled corticosteroid, LABA = long-acting beta-agonist, LAMA = long-acting muscarinic antagonist.

* Medication used alone or in combination.

**Figure 2.**
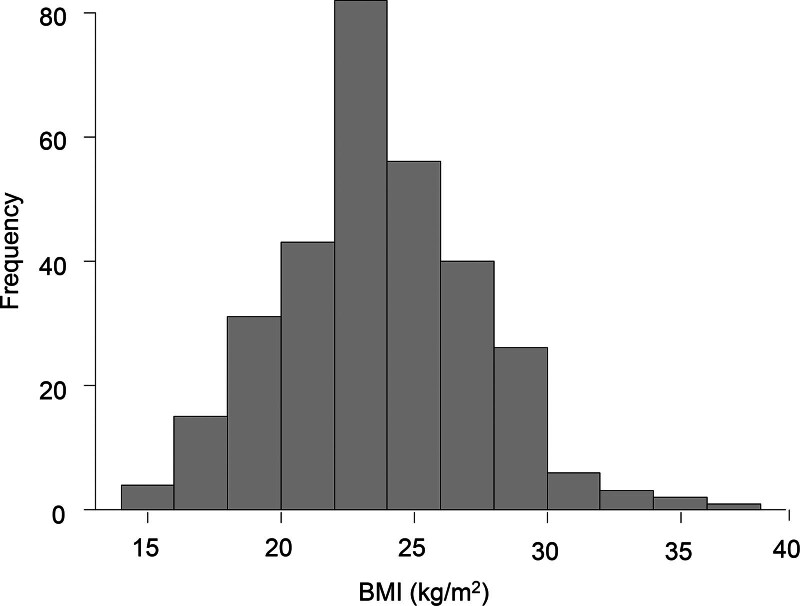
Body mass index values of the study participants. This figure shows the distribution of BMI values in malnourished and well-nourished patients with COPD. BMI, body mass index; COPD, chronic obstructive pulmonary disease.

The distribution of BMI values is shown in Figure 2. The mean BMI was 23.7 kg/m², ranging from 15.1 to 37.4 kg/m². Among the 309 patients, 45 (14.6%) were diagnosed with malnutrition. The prevalence of malnutrition according to GOLD stages 1, 2, 3, and 4 was 6.1% (4 of 66), 11.3% (17 of 151), 17.7% (11 of 62), and 43.3% (13 of 30), respectively. The prevalence of malnutrition according to GOLD ABE was as follows: A, 9.2% (19 of 207); B, 20.8% (15 of 72); and E, 36.7% (11 of 30).

Of the 309 patients, 275 (89%) completed the 5-year follow-up, and mean follow-up time was 4.3 years. The final body weight measurements were obtained for 201 patients. Median change of body weight was −0.54 kg (IQR, −1.12 to 0.08) per year from baseline. There was no difference in annual body weight change between the patients with and without malnutrition (−0.66 ± 0.98 kg vs −0.52 ± 0.93 kg; *P* = .54). No patient exhibited >5% weight loss per year from baseline.

### 3.2. Comparison between patients with or without malnutrition

The characteristics of patients with and without malnutrition are presented in Table 2.

**Table 2:** Comparison of characteristics of patients with COPD with or without malnutrition.

Characteristic	Malnutrition (N = 45)	No malnutrition (N = 264)	*P*-value
Age, years	77.2 ± 6.8	74.8 ± 7.2	.038
Age category, years			.15
40–64	3 (6.7)	18 (6.8)	
65–74	11 (24.4)	105 (39.8)	
75–84	25 (55.6)	122 (46.2)	
≥85	6 (13.3)	19 (7.2)	
Sex			>.99
Male	43 (95.6)	251 (95.1)	
Female	2 (4.4)	6 (4.9)	
BMI, kg/m²	18.1 ± 1.3	24.6 ± 3.0	<.001
Smoking history, pack-years	63.4 ± 27.9	55.2 ± 25.4	.049
Current smoking	1 (2.2)	12 (4.5)	.70
FEV_1_, L	1.18 ± 0.56	1.61 ± 0.59	<.001
%FEV_1_, %	48.2 ± 23.0	63.3 ± 20.1	<.001
FVC, L	2.78 ± 0.76	3.12 ± 0.78	.0073
GOLD stage			<.001
1	4 (8.9)	62 (23.5)	
2	17 (37.8)	134 (50.8)	
3	11 (24.4)	51 (19.3)	
4	13 (28.9)	17 (6.4)	
GOLD group			<.001
A	19 (42.2)	188 (71.2)	
B	15 (33.3)	57 (21.6)	
E	11 (24.4)	19 (7.2)	
Regular medication*			
LAMA	37 (82.2)	218 (82.6)	>.99
LABA	33 (73.3)	169 (64.0)	.24
ICS	10 (22.2)	75 (28.4)	.47
Long-term oxygen therapy	10 (22.2)	24 (9.1)	.017
Comorbidity			
Cardiovascular disease	11 (24.4)	86 (32.6)	.30
Cerebrovascular disease	2 (4.4)	17 (6.4)	>.99
Diabetes	5 (11.1)	41 (15.5)	.65
Gastroduodenal ulcer or GERD	5 (12.2)	33 (12.5)	>.99
Osteoporosis	8 (17.8)	28 (10.6)	.20
mMRC	1 (0–3)	0 (0–1)	<.001
CAT	6 (3–12)	3 (1–7)	<.001
Depression§	6 (3–9)	4 (1–7)	.024
Anxiety§	4 (2–6)	3 (1–6)	.23
Handgrip strength, kg‡	27.7 ± 6.8	32.9 ± 8.0	<.001
Muscle weakness‡	23 (52.3)	67 (25.6)	<.001
6-minute walk distance, m∥	287 ± 126	352 ± 113	<.001
Daily step counts, steps	3286 ± 3304	4980 ± 3425	.0022
CRP, mg/dL	0.32 ± 0.71	0.30 ± 0.54	.83

Data are presented as numbers (percentages), mean ± SD, or median (interquartile range).

BMI = body mass index, CAT = COPD Assessment Test, CRP = C-reactive protein, FEV_1_ = forced expiratory volume in 1 second, FVC = forced vital capacity, GERD = gastroesophageal reflux disease, GOLD = global initiative for chronic obstructive lung disease, ICS = inhaled corticosteroid, LABA = long-acting beta-agonist, LAMA = long-acting muscarinic antagonist, mMRC = modified Medical Research Council, SD = standard deviation.

* Medication used alone or in combination.

§ One patient with malnutrition and 1 patient without malnutrition did not complete the Hospital Anxiety and Depression Scale.

‡ One patient with malnutrition and 2 patients without malnutrition did not undergo handgrip strength evaluation.

∥ Two patients with malnutrition and 1 patient without malnutrition declined the 6-minute walk test due to dyspnea.

Patients with malnutrition were older and had a greater smoking history (both *P* < .05). They exhibited more severe airflow obstruction and lower exercise capacity (both *P* < .001). Additionally, they experienced increased dyspnea and muscle weakness (all *P* < .001), as well as depressive state, poorer health status (both *P* < .05), and physical inactivity (*P* < .01).

No significant differences were observed between the groups in terms of sex, current smoking status, regular medication use (except for long-term oxygen therapy), comorbidities, anxiety, or C-reactive protein levels.

### 3.3. Exacerbations of patients with COPD and malnutrition

Among the 309 patients, 307 (99%) completed 1 year of follow-up. During the study period, patients with malnutrition experienced more frequent exacerbations overall, as well as more severe exacerbations requiring hospital admission, compared with those without malnutrition (Table 3). Patients with malnutrition had an odds ratios of 2.7 (95% CI, 1.2–5.9) and 3.6 (95% CI, 1.5–8.7) for an increased risk of exacerbations and hospital admission, respectively, adjusted for age, smoking history, and GOLD staging.

**Table 3:** Comparison of exacerbations in patients with or without malnutrition.

Outcomes	Malnutrition (N = 44)	No malnutrition (N = 263)	*P*-value
All exacerbations			
No. (%)	17 (38.6)	35 (13.3)	<.001
Annual rate	0.55 ± 0.87	0.18 ± 0.54	.010
Hospital admission			
No. (%)	14 (31.8)	18 (6.8)	<.001
Annual rate	0.39 ± 0.65	0.099 ± 0.43	.0069

The data are presented as numbers (percentages) or mean ± SD.

SD = standard deviation.

### 3.4. Mortality of patients with COPD and malnutrition

Following a 5-year observation period, 23% (62 of 275) of patients died due to respiratory-associated events, including exacerbation and pneumonia (n = 10), cancer (n = 14), cardiovascular disease (n = 6), stroke (n = 5), and other diseases or unknown (n = 27).

All-cause mortality was significantly higher in patients with malnutrition compared with those without malnutrition (43.6% vs 19.1%, *P* = .0015) (Table 4). Similarly, respiratory-related mortality was also higher in patients with malnutrition than in those without malnutrition (10.3% vs 2.5%, *P* = .0038).

**Table 4:** Comparison of 5-year mortality in patients with or without malnutrition.

Outcomes	Malnutrition (N = 39)	No malnutrition (N = 236)	*P*-value
All-cause mortality	17 (43.6)	45 (19.1)	.0015
Respiratory-associated mortality	4 (10.3)	6 (2.5)	.0038

The data are presented as numbers (percentages).

**Figure 3.**
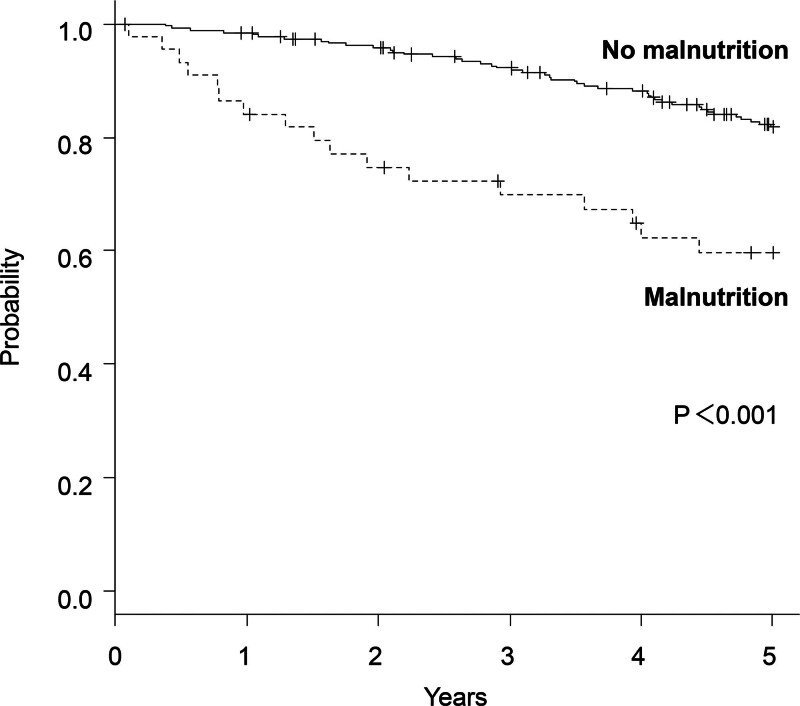
Kaplan–Meier curves for survival, stratified by diagnosis of malnutrition. This figure illustrates the survival probabilities of patients with COPD with malnutrition versus those without malnutrition over a 5-year period. The curves indicate significantly lower survival rates in malnourished patients (*P* < .001). COPD, chronic obstructive pulmonary disease.

The Kaplan–Meier survival curves are shown in Figure 3. Patients without malnutrition demonstrated a survival advantage over those with malnutrition (*P* < .001). In a Cox proportional hazard model, adjusted for confounders, all-cause mortality was significantly associated with malnutrition (Table 5).

**Table 5:** Adjusted hazard ratios for 5-year all-cause mortality.

	Hazard ratio	95% Confidence interval
Model 1	2.3	1.3–4.2
Model 2	2.0	1.1–3.5
Model 3	2.2	1.2–3.9

Model 1: Adjusted for age, sex, smoking history, and %FEV_1_.

Model 2: Adjusted for age, sex, smoking history, and mMRC.

Model 3: Adjusted for age, sex, smoking history, and daily step counts.

FEV_1_ = forced expiratory volume in 1 second, mMRC = modified Medical Research Council.

## 4. Discussion

This prospective observational study investigated the impact of malnutrition, defined by low BMI according to the GLIM criteria, in a cohort of Japanese patients with COPD.

To diagnose malnutrition in at-risk patients, patients should be identified using a validated screening tool and evaluated according to the established diagnostic criteria. Previous studies misused nutrition screening tools, such as short-form mini-nutritional assessment^[19]^ and Nutritional Risk Screening 2002,^[20,21]^ to diagnosis malnutrition. Moreover, various diagnostic criteria were used, such as Subjective Global Assessment^[22,23]^ and consensus criteria of the European Society of Clinical Nutrition and Metabolism.^[24,25]^ The GLIM criteria were published in 2019 and have since been implemented in clinical settings worldwide.^[9,10]^ However, data on their application in COPD remain limited, with only a few studies available, such as a small-sample study involving 16 patients with COPD^[26]^ and an observational study using the Japanese medical claims database.^[27]^

We demonstrated that malnutrition was associated with decreased respiratory function, increased dyspnea, reduced exercise capacity, worsened quality of life, and physical inactivity in COPD, consistent with findings from previous studies.^[3,6]^ Moreover, the findings of our study revealed that malnutrition was associated with higher rates of exacerbations, including hospital admissions, as well as increased 5-year all-cause mortality in COPD, suggesting an association between malnutrition and poor outcomes across different settings and study populations.^[8]^

In addition, our findings indicated that malnutrition was associated with muscle weakness. Malnutrition can lead to a negative skeletal muscle protein balance, resulting in muscle loss.^[28]^ Muscle weakness and muscle loss are hallmark symptoms of sarcopenia, which is characterized by low muscle mass and reduced muscle function (e.g., low handgrip strength or slow gait speed) that commonly occur with advanced age.^[29,30]^ Although malnutrition and sarcopenia are distinct conditions, they share underlying causes, including decreased food intake, age-related physiological changes, and systemic inflammation; therefore, the 2 conditions frequently coexist. In this study, 52% of malnourished patients exhibited muscle weakness, suggesting the possible presence of sarcopenia. These findings indicate that malnutrition contributes to decreased muscle mass and reduced muscle strength, resulting in impaired respiratory function, increased dyspnea, reduced exercise capacity, worsened quality of life, and physical inactivity, which leads to frequent exacerbations and increased mortality in patients with COPD.

In this study, the prevalence of malnutrition was 15% among community-dwelling, stable outpatients with COPD. Previous studies reported that the prevalence of malnutrition in patients with COPD was ~30%, with a wide range (3.7%–83%) depending on clinical setting and disease severity.^[4]^ Additionally, 44% (14 of 32) of patients who required hospital admission in this study had malnutrition, which aligns with findings from a previous observational study conducted in Japan.^[27]^

This study has some limitations. First, malnutrition diagnosis was based solely on low BMI from the GLIM and did not include the other 2 phenotypic criteria (non-volitional weight loss and reduced muscle mass). Patients with a normal BMI but non-volitional weight loss or reduced muscle mass may have had their malnutrition underestimated. Non-volitional weight loss is defined as a weight loss of >5% within the past 6 months or >10% beyond 6 months.^[9]^ In this study, the annual body weight change was −0.54 kg in patients with stable COPD, and no patients experienced >5% weight loss per year. Our observations align with findings from another cohort study in Japanese patients with COPD,^[31]^ suggesting that non-volitional weight loss rarely occurs in Japanese patients with stable COPD. Although we could not assess body weight change within the past 6 months at baseline, it had a limited impact on the potential underdiagnosis of malnutrition. Moreover, to evaluate reduced muscle mass, the GLIM recommends measurement using dual-energy X-ray absorptiometry or other validated body composition techniques^[8]^; however, thresholds for reduced muscle mass have not been established for Asian populations. Therefore, this criterion could not be applied in our study. Further research is warranted to investigate the effectiveness of body composition analysis for diagnosing malnutrition in Japanese patients with COPD. Second, this study did not assess actual dietary intake and socioeconomic status, which were associated with nutritional status in patients with COPD.^[32,33]^ The possibility of impacts on these unmeasured confounders cannot be ruled out. Third, this cohort consisted predominantly of male patients (over 95%), which limits the generalizability of the findings to female patients with COPD. Additionally, the inclusion of only smokers may be considered a limitation, as never-smoker patients with COPD were not represented.

Previous studies demonstrated that patients with low BMI showed higher mortality compared with patients having a normal BMI.^[34,35]^ It is imperative to not only acknowledge the prevalence of underweight but also accurately diagnose malnutrition following interventions in patients suffering from COPD. Early diagnosis and treatment of malnutrition can improve clinical outcomes in these patients. Although nutritional support has not been consistently shown to improve lung function and mortality, dietary advice and oral supplementation have been reported to enhance body weight, respiratory muscle strength, and quality of life.^[3,36,37]^

Nutritional evaluation process has not been established and routine nutritional screening is not standardized in patents with COPD. We propose that all patients with COPD should undergo nutritional evaluation, including assessment of body weight, dietary habits, food intake, and mealtime-related symptoms, and patients diagnosed with malnutrition according the GLIM criteria should receive personalized dietary guidance, including recommendations on energy and protein requirements, supplementation with micronutrients and trace elements, and oral nutritional supplements, if necessary. Notably, early detection and treatment of malnutrition may be particularly essential for patients with severe disease (GOLD 4 and E) and those who are hospitalized.

## 5. Conclusions

We conducted a prospective observational study to evaluate the prevalence, characteristics, and outcomes of malnutrition according to low BMI of the GLIM criteria in a cohort of Japanese patients with COPD. The results demonstrated that malnutrition was associated with older age, greater smoking history, more severe airflow obstruction, increased dyspnea, poorer health status, depressive state, muscle weakness, reduced exercise capacity, and physical inactivity. Moreover, this study showed that malnutrition was associated with higher rates of exacerbations, including hospital admissions, and increased 5-year all-cause mortality. These findings underscore the importance of routine malnutrition screening in patients with COPD and highlight the potential for nutritional interventions to reduce exacerbations and improve survival outcomes.

## Acknowledgments

We would like to thank all the expert nurses at the Outpatient Clinic of the Japanese Red Cross Ishinomaki Hospital for their exceptional patient care and assistance with data acquisition. We are also grateful to the healthcare professionals affiliated with ICON for their invaluable support and cooperation in this research. We would like to thank Editage (www.editage.jp) for English language editing.

## Author contributions

**Conceptualization:** Seiichi Kobayashi.

**Investigation:** Seiichi Kobayashi, Manabu Ono, Masatsugu Ishida, Hikari Satoh, Masakazu Hanagama, Masaru Yanai.

**Data curation & Formal analysis:** Seiichi Kobayashi.

**Resources:** Seiichi Kobayashi, Manabu Ono, Masatsugu Ishida, Hikari Satoh, Masakazu Hanagama, Masaru Yanai.

**Supervision:** Masaru Yanai.

**Writing – original draft:** Seiichi Kobayashi.

**Writing – review & editing:** Seiichi Kobayashi, Manabu Ono, Masatsugu Ishida, Hikari Satoh, Masakazu Hanagama, Masaru Yanai.
